# The First Examples of [3+2] Cycloadditions with the Participation of (*E*)-3,3,3-Tribromo-1-Nitroprop-1-Ene

**DOI:** 10.3390/ma15217584

**Published:** 2022-10-28

**Authors:** Karolina Zawadzińska, Zuzanna Gadocha, Kamila Pabian, Aneta Wróblewska, Ewelina Wielgus, Radomir Jasiński

**Affiliations:** 1 Department of Organic Chemistry and Technology, Cracow University of Technology, Warszawska 24, 31-155 Cracow, Poland; 2 Centre of Molecular and Macromolecular Studies, Polish Academy of Sciences, Sienkiewicza 112, 90-363 Lodz, Poland

**Keywords:** [3+2] cycloaddition, 3,3,3-tribromo-1-nitroprop-1-ene, nitrones, molecular mechanism, molecular electron density theory

## Abstract

The first examples of [3+2] cycloaddition reactions between 3,3,3-tribromo-1-nitroprop-1-ene (TBMN) were explored on the basis of experimental and theoretical approaches. It was found that reactions involving TBMN and diarylnitrones realized with full regio- and stereoselectivity lead to respective 3,4-*cis*-4,5-*trans*-4-nitroisoxazolidines. The regioselecticity and the molecular mechanism of title processes was analyzed on the basis of the advanced DFT computational study.

## 1. Introduction

It is generally known that the trihalomethyl CX_3_ (THM) functional group is an important element of many bioactive structures [1,2,3]. The possibility of the easy and selective introduction of this type groups into heterocyclic systems and exhibits [3+2] cycloaddition reactions involving TMH-functionalized alkenes [4,5,6]. Including the same alkene system, both TMH as well as the nitrogroup give an attractive channel for the further potential functionalization of target molecules [7]. So, TMH substituted conjugated nitroalkenes attract the attention of various research centers. In particular, 3,3,3-trichloro-1-nitroprop-1-ene was recently intensively tested as a component of different type cycloadditions involving diazocompounds [8,9], nitrile *N*-oxides [10], and especially nitrones [11,12,13,14]. Regarding 3,3,3-trifluor-1-nitroprop-1-ene, many interesting scientific works are also available at the present time [4,5,6,15,16]. In contrast, only some incidential contributions regarding the physicochemistry of 3,3,3-tribromo-1-nitroprop-1-ene (TBMN) are available [17,18]. In particular, only one simple example of [4+2] cycloaddition with the participation of TBMN and simple aliphatic dienes is known [17]. Any other examples of 32CAs of TBMN were not described for this time. Due to the issues mentioned above, within this work we initiated systematic studies in the area of cycloaddition reactions with the participation of TBMN. In particular, we decided to explain the reaction course of model 32CAs between series of *Z-C*-aryl-*N*-phenylnitrones (**1a–g**) and TBMN (**2**). In the framework of our research, we analyzed (i) regio- and stereochemical aspects of these reactions under experimental conditions and (ii) the mechanistic aspect of observed reaction channels based on results of DFT computational study. We hope these studies will be helpful in understanding the role of the CBr_3_ group in the cycloaddition process and in enriching the knowledge of TBNM reactivity.

## 2. Results and Discussion

**Scheme 1:**
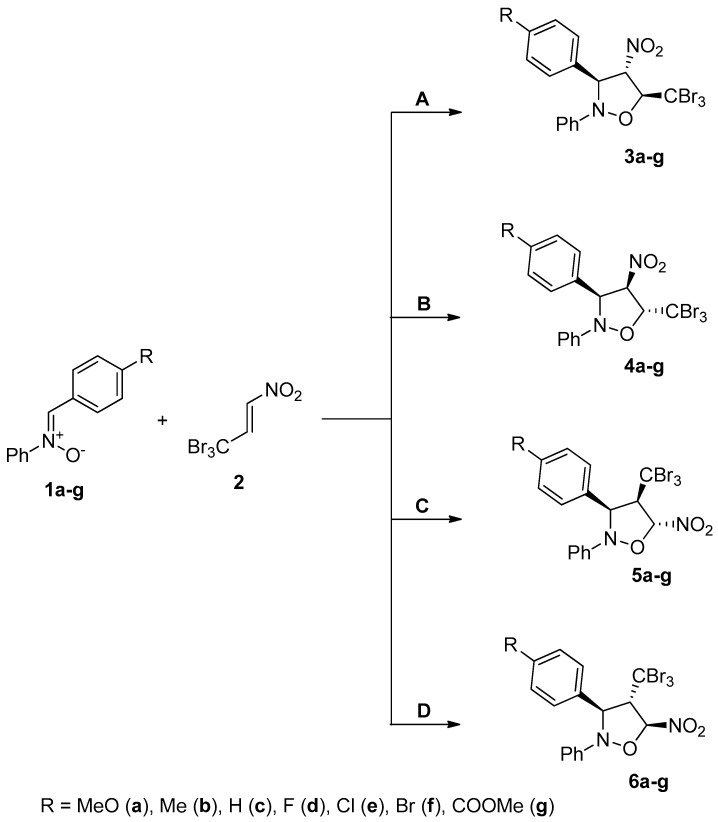
Theoretical possible paths of 32CAs of *Z-C*-aryl-*N*-phenylnitrones (**1a–g**) and TBMN (**2**).

Examined reactions, assuming their one-step cycloaddition mechanism [19], can proceed via four independent regio- and stereochemical channels, leading to respective nitrosubstituted 2,3-diarylisoxazolidines (Scheme 1).

Our research started from the reaction involving nitrone **1c**. During the search for acceptable conditions of the cycloaddition process, we performed tests under different temperatures, molar ratio of reagents, and solvents as well as reaction times. The reaction progress was checked using the HPLC system. It was found that the cycloaddition [3+2] proceeded easily at r.t. in benzene with the reagent molar ratio 1:2. After 17 h the conversion of addents was completed. HPLC analysis confirmed the presence of only one product in the postreaction mixture. This compound was isolated using crystallization from ethanol and identified on the basis of spectral techniques. In particular, absorption bands typical of the nitro group [20], C-Br bonds [21] and isoxazolidine ring [13] were identified in the IR spectrum. Then, we did a high resolution mass spectrum obtained by the atmospheric pressure chemical ionization technique. The APCI mass spectrum is characterized by protonated molecular ion and a few prominent fragment ions. The elemental composition of protonated molecular ion with m/z 518.8565 confirmed the molecular formula C_16_H_14_N_2_O_3_Br_3_.

The major fragment ions (m/z values and molecular formulas) are summarized. One of the fragmentation peaks which corresponds to the ion formed by elimination of C_2_HOBr_3_ allowed us to assume that the cycloaddition reaction may lead to isomers **3c** or **4c** (nitro group positioned in 4). Unfortunately, assignment of the stereochemistry is not possible based only on mass spectrometry analysis.

Further data was obtained from the NMR spectra. In particular, the product obtained via reaction **1c+2** was selected as a model to determine the configuration of the obtained products. The HSQC spectrum shows a correlation between doublet of doublets signal of the proton with the carbon C(4) located at 95.8 ppm, while carbon C(3) is located at 74.8 ppm, and C(5) is at 89.5 ppm. The HMBC ^1^H-^13^C spectrum provides more information about the localization of the CBr_3_ and phenyl groups. Both carbons C(3) and C(5) show the correlation with a proton from C(4), but only carbon C(3) shows the correlation with protons from the phenyl group. It was confirmed that the phenyl moiety is attached directly to C(3). On the other hand, the same spectrum shows only two correlations of carbon CBr_3_ located at 37.5 ppm, with protons bonded with C(4) and C(5). Due to non existence of the third correlation, the CBr_3_ moiety is located directly with carbon C(5). Therefore, the nitro group must be in the C(4) position. On the ^1^HNMR spectrum of the considered structure, two doublets and one doublet of doublets are observed in the area characteristic for isoxazolidine ring protons. The HSQC spectrum shows very clearly, that doublet of doublets corresponding to one proton at 5.60 ppm with JH-H = 8.4 Hz and JH-H = 4.7 Hz belong to carbon C(4). The first doublet corresponds to one proton at 5.80 ppm correlated with C(5). Its J-coupling is equal to the value JH-H = 4.7 Hz, which is very characteristic for *trans* position between two substituents in similar isoxazolidine, in this case, between nitro moiety from C(4) and CBr_3_ located at C(5). The second doublet corresponds to one proton at 5.05 ppm correlated with C(3). The J-coupling is equal to JH-H = 8.3 Hz and is a characteristic of the *cis* position for substituents on the isoxazolidine ring.

To summarize, the configuration of 3,4-*cis*-4,5-*trans*-2,3-diphenyl-4-nitro-5-tribromomethylisoxazolidine **4c** can be assigned. In a similar way, we analyzed all reactions with nitrones **1a–g**. In all cases, only respective 3,4-*cis*-4,5-*trans*-2-phenyl-3aryl-4-nitro-5-tribromomethylisoxazolidines were isolated as single cycloaddition products. It was established that independently of the substituents’ nature in the nitrone molecule, these 32CAs with the participation of TBNM are realized with full regio- and stereoselectivity. It is interesting that similar 32CAs involving 1-nitroprop-1-ene proceed without full stereoselectivity and lead to the mixture of 3,4-*cis*- and 3,4-*trans* isomers with the ratio 5.7:1 [22]. This suggest the important influence of the volume of the substituent at β-position of nitrovinyl moiety on the reaction stereoselectivity.

The regioselectivity of the reactions can be easy to explain in the framework of Molecular Electron Density Theory [23]. This approach has been successfully applied for the explanation of a number of bimolecular reactions [24,25,26,27], including [3+2] cycloadditions. It was found that the global electrophilicity of the nitroalkene **2** is equal to 3.23 eV. Within the unique electrophilicity scale, this component should be treated as an evidently electrophilic component. On the other hand, the global electrophilicity of benzonitrile N-oxide is equal to only 1.67 eV. This value is typical for moderate electrophiles. It should be noted here that the replacement of hydrogen atom in the benzene moiety of N-oxide to the electron-donating groups stimulate the further decreasing of the electrophilic properties. On the other hand, the introduction of an electron-withdrawing group stimulates increase of the ω values. However, in all cases, **2** is a more electrophilic agent than the second component. So, global properties of N-oxides **1a–g** should be fully characterized by using global nucleophilicity indices. All compounds from this group exhibit global nucleophilicity in the range of 3.39–3.98 eV. In conclusion, all considered processes should be treated as evidently polar, and for the interpretation of the courses, the analysis of local nucleophile–electrophile interactions can be applied. In particular, it was found that the most nucleophilic reaction center at all nitrones >C=N(O)- moieties is always located on the oxygen atom (1.47–1.64 eV) (Table 1). On the other hand, the most electrophilic center at the nitrovinyl moiety of the nitroalkene is assigned with the β-carbon atom (0.71 eV). The interactions of the mentioned reaction centers must lead to the formation of respective 4-nitroisoxazolidines.

**Figure 1:**
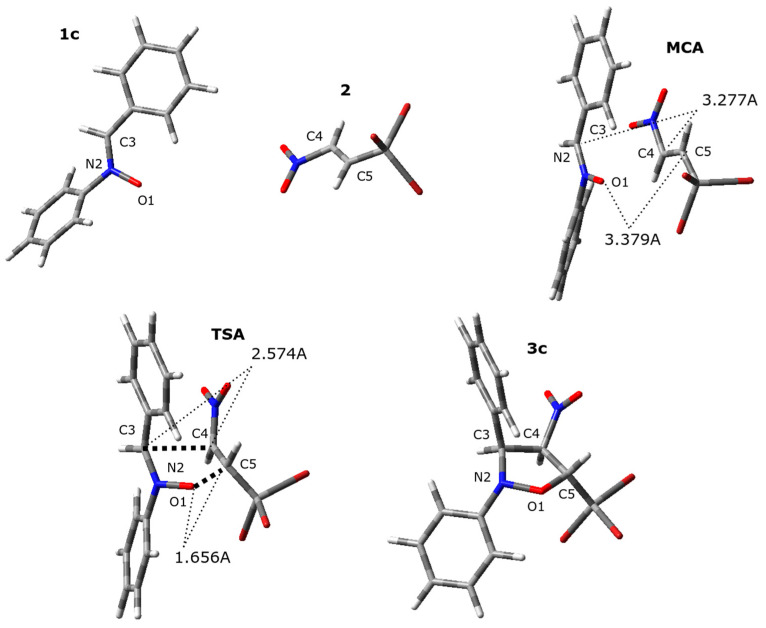
Views of critical structures for reaction **1c+2→3c** in light of the wb97xd/6-311+G(d) (PCM) calculations.

At the end, we performed the exploration of reaction profiles for the better understanding of the cycloaddition nature. For this purpose, the data obtained from the DFT wb97xd/6-311+G(d) (PCM) computational study was applied (Table 2 and Table 3). For the resolving of mechanistic aspects of different-type pseudocyclic organic reactions, a similar level of the theory was used [28,29,30]. The DFT calculations for the model cycloaddition **1c+2** shows that the enthalpy and Gibbs free energy factors are favoring the formation of 3,4-cis-3,4-trans cycloadduct, which was isolated from the postreaction mixture. Next, we decided to examine the molecular mechanism of the formation of a heterocyclic ring on the experimentally observed reaction path. It was found that the conversion of addents into a target **4c** molecule is realized via two critical points. Firstly, the pre-reaction molecular complex (MC) is formed. This is accompanied by the reduction of the enthalpy of the reaction system by 9 kcal/mol. Within MC, both substructures adopt the orientation, which determine the further direction of the new bond formation. Subsequently, any new sigma bonds are not formed at this stage. Additionally, MC should not be considered as a charge-transfer complex because the GEDT indice is equal to 0.00e. The subsequent transformation of the MC leads to the area of transition state (TS). This stage requires the enthalpy of the activation, which is equal to 4.9 kcal/mol. Within TS, key interatomic distances are essentially decreased to 2.574 Å and 1.656 Å (C3-C4 and C5-C5), respectively (Figure 1). Therefore, TS should be considered as evidently asynchronous. Additionally, it exhibits a clearly polar nature, which is confirmed by great GEDT value (0.66e). The detailed analysis of the IRC trajectories show, due to Domingo and Rios-Gutierrez terminology [19], that the analyzed process should be interpreted as “one step cycloaddition”.

## 3. Methods and Procedures

### 3.1. Experimental

#### 3.1.1. Analytical Techniques

HPLC analyses were done using a Knauer UV VIS detector (LiChrospher 18-RP 10 µm column, eluent: 80% methanol). M.p. were estimated on the Boetius apparatus and were uncorrected. IR spectra were derived from the FTS Nicolet IS 10 spectrophotometer. ^1^HNMR spectra were recorded on an AV 400 Neo spectrometer or on a Bruker Avance III 600 spectrometer and are reported in ppm using deuterated solvent as an internal standard (CDCl_3_ at 7.26 ppm). Data were reported as s = singlet, d = doublet, dd = doublet of doublets, m = multiplet. ^13^CNMR spectra were recorded on an AV 400 Neo 101 MHz spectrometer or on a Bruker Avance III 151 MHz spectrometer and were reported in ppm using deuterated solvent as an internal standard (CDCl_3_ at 77.2 ppm). ^19^FNMR spectrum was recorded on an AV 400 Neo spectrometer and reported in ppm using deuterated solvent as an internal standard CDCl_3_. High-resolution mass spectrometry (HRMS) measurements were performed using Synapt G2-Si mass spectrometer (Waters, Milford, MA, USA) equipped with an atmospheric pressure chemical ionization (APCI) source and quadrupole Time-of-Flight mass analyzer. The mass spectrometer was operated in the positive ion detection mode with a discharge current set at 4.0 μA. The heated capillary temperature was 350 °C. To ensure accurate mass measurements, data were collected in centroid mode and mass was corrected during acquisition using leucine enkephalin solution as an external reference (Lock-SprayTM), which generated reference ion at m/z 556.2771 Da ([M+H]^+^) in positive APCI mode. The results of the measurements were processed using MassLynx 4.1 software (Waters) incorporated with the instrument.

#### 3.1.2. Materials

The components of the 32CA were synthesized in accordance with procedures described in the literature. In particular, *Z-C*-aryl-*N*-phenylnitrones (**1a–g**) were prepared via condensation between *N*-phenylohydroxylamine and respective arylaldehydes [31]. The 3,3,3-bromo-1-nitroprop-1-ene (**2**) was obtained via three-step method, starting from nitromethane and bromal (see Supplementary Material for the details) [17,18]. Commercially available (Sigma Aldrich, St. Louis, MO, USA) chemicals have been used as solvents and components for the further synthesis of addends for considered 32CA.

#### 3.1.3. Cycloaddition between *Z-C*-aryl-*N*-Phenylnitrones (**1a–g**) and TBMN (**2**) General Procedure

A solution of 3,3,3-bromo-1-nitroprop-1-ene (0.02 mol) and appropriate nitrone (0.01 mol) in dry benzene (25 mL) was mixed at room temperature for 24 h. The post-reaction mixture was filtered, and the solvent was evaporated in vacuo. The isolation of the reaction products from the post-reaction mixtures was performed via crystallization from ethanol. Pure products were identified on the basis of HR-MS, IR and NMR spectral data.

### 3.2. Computational Details

The quantumchemical calculations reported in this paper were performed using wb97xd functional with the 6-311+G(d) basis set included in the GAUSSIAN 09 package [32]. Optimizations of the critical structures were performed with the Berny algorithm, whereas the transition states (TSs) were calculated using the QST2 procedure. Subsequently, TSs on considered paths were localized via alternative methodology by gradually changing the distance between reaction centers (with optimization after each step). It should be underlined that in this way TSs identical as previously have been obtained. Localized critical points were successfully verified by frequency calculations. It was found that all reactants and products were characterized by positive Hessian matrices. Subsequently, all TSs showed only one negative eigenvalue in their diagonalized Hessian matrices. Next, their associated eigenvectors were confirmed to correspond to the motion along the reaction coordinate under consideration. For further verification of TSs, IRC calculations were performed. The solvent effect on the reaction paths was included using the polarizable continuum model (PCM) [33]. Global electron density transfer between substructures (GEDT) [34] was calculated according to the equation GEDT = Σq_A_ where q_A_ is the net charge and the sum is taken over all the atoms of nitroalkene. New σ-bonds development (l) was expressed in the correlation to distance between the reaction centers in the transition structure (rTSX-Y) and the same distance in the corresponding product (rPX-Y) [35]: $l_{X-Y}=1-\frac{r_{X-Y}^{\mathrm{TS}}-r_{X-Y}^{P}}{r_{X-Y}^{P}}$. Electronic properties of reactants were estimated according to recommended earlier relationships [36,37]. ω = μ^2^/2η, μ ≈ (E_HOMO_ + E_LUMO_)/2, η ≈ E_LUMO_ − E_HOMO_. Global nucleophilicities (N) [38] were calculated using equation: N = E_HOMO_ − E_HOMO (tetracyanoethene)._ The local electrophilicity (ω_k_) on the atom k was calculated using index ω and respective *Parr* function P_+k_ [39]: ω_k_ = P^+^_k_·ω. The local nucleophilicity (Nk) on the atom k was calculated using index N and *Parr* respective function P^-^_k_ [39]: N_k_ = P^-^_k_·N.

## 4. Conclusions

This experimental and theoretical studies shed light on the course of the unique examples of [3+2] cycloaddition reactions with the participation of the very poorly known 3,3,3-bromo-1-nitroprop-1-ene. In particular, [3+2] cycloadditions between mentioned nitroalkene and diarylnitrones realize with full regio- and stereoselectivity and lead to respective 3,4-*cis*-4,5-*trans*-4-nitroisoxazolidines. So, the 32CA selectivity is fundamentally different than other similar processes with the participation of diarylnitrones and conjugated nitroalkenes. Additionally, the DFT wb97xd/6-311+G(d) (PCM) shows without any doubt that all explored processes should be considered as polar and are realized via one step.

## Figures and Tables

**Table 1:** Global and local electronic properties of diarylnitrones **1a–g** and TBMN **2**.

	R	ω [eV]	N [eV]	P^-^_C_	P^-^_O_	N_C_ [eV]	N_O_ [eV]	P^+^_α_	P^+^_β_	ω_α_ [eV]	ω_β_ [eV]
**1a**	OMe	1.48	3.98	0.04	0.41	0.16	1.62				
**1b**	Me	1.60	3.77	0.10	0.44	0.38	1.64				
**1c**	H	1.67	3.64	0.14	0.44	0.51	1.61				
**1d**	F	1.71	3.60	0.12	0.43	0.44	1.56				
**1e**	Cl	1.87	3.48	0.12	0.42	0.41	1.47				
**1f**	Br	1.88	3.49	0.11	0.41	0.37	1.45				
**1g**	COOMe	2.11	3.39	0.15	0.43	0.50	1.47				
**2**	-	3.23	1.09					0.13	0.22	0.41	0.71

**Table 2:** Key parameters for critical structures for reaction **1c+2→3c** in in light of the wb97xd/6-311+G(d) (PCM) calculations.

Structure	Interatomic Distances [Å]					l_C3-C4_	l_C5-O1_	Δl	GEDT [e]
	O1-N2	N2-C3	C3-C4	C4-C5	C5-O1				
**1c**	1.276	1.307							
**2**				1.322	...				
**MCA**	1.276	1.307	3.277	1.323	3.379				0.00
**TSA**	1.332	1.315	2.574	1.424	1.656	0.353	0.822	0.47	0.66
**3c**	1.407	1.474	1.563	1.530	1.405				

**Table 3:** Energetical parameters for reaction **1c+2** in light of the wb97xd/6-311+G(d) (PCM) calculations.

Transition	ΔH	ΔG	ΔS
**1c+2** **→LMA**	−9.0	3.8	−43.2
**1c+2** **→TSA**	6.0	22.0	−53.5
**1c+2** **→3c**	−27.5	−11.6	−53.5
**1c+2** **→LMB**	−8.3	4.0	−41.1
**1c+2** **→TSB**	4.9	20.2	−51.5
**1c+2** **→4c**	−31.7	−15.8	−53.1
**1c+2** **→LMC**	−8.5	3.9	−41.6
**1c+2** **→TSC**	8.5	24.0	−51.9
**1c+2** **→5c**	−29.6	−13.2	−55.1
**1c+2** **→LMD**	−10.3	3.6	−46.6
**1c+2** **→TSD**	12.8	28.8	−53.9
**1c+2** **→6c**	−26.4	−10.2	−54.5

## Data Availability

The data presented in this study are available on request from the corresponding author.

## Supplementary Materials

The following supporting information can be downloaded at: https://www.mdpi.com/article/10.3390/ma15217584/s1, Table S1. The m/z values and a molecular formula of the major fragment ions in APCI mass spectra of **4a–4g**.

## Sample Availability

Samples of the compounds are not available from the authors.
