# Supplementary material for: The First Examples of [3+2] Cycloadditions with the Participation of (E)-3,3,3-Tribromo-1-Nitroprop-1-Ene
Source: Materials (Basel). 2022 Oct 28;15(21):7584. doi: 10.3390/ma15217584 (PMC9657535; doi:10.3390/ma15217584)
Supplement: Supplementary file 1 [file materials-15-07584-s001.zip › materials-1978895-supplementary.pdf]

SUPPLEMENTARY MATERIAL

First examples of [3+2] cycloadditions with the participation of the (E)-3,3,3-tribromo-1-nitroprop-1-ene

Karolina Zawadzińska, Zuzanna Gadocha, Kamila Pabian, Aneta Wróblewska,

Ewelina Wielgus, Radomir Jasiński

[1] Synthetic procedures

[1.1.] 1,1,1-tribromo-3-nitropropan-2-ol

Nitromethane 0.125 mol (6.75 ml) was added upon stirring to a solution of 0.084 mol (25 g) of bromal in 40 ml of water. After dosing of nitromethane to the bromal solution, the reaction mixture was heated to 45-50°C. During this time 1.05 g of K_2CO_3 was then added in portions to the reaction mixture. When temperature reached 50°C, the mixture was stirred for another 30 minutes. After that the reaction mixture was neutralized with dilute hydrochloric acid. The resulting nitro alcohol was separated off by extraction with chloroform (6 × 25 ml). The following chloroform extracts were combined and dried over $CaCl_2$. Chloroform was evaporated in vacuum. Obtained colorless oil was crystallizing immediately mp. 75–77°C. Yielded 21,2 g (74%).

[1.2.] 1,1,1-tribromo-3-nitro-2-acetoxypropan

Acetyl chloride 0.124 mol (8.85 ml) was added to obtained 0.068 mol (21.2 g) nitro alcohol. The reaction mixture was heated at a water bath to 77°C untill dissolving of the nitro alcohol and then incubated for 12 h at room temperature. Excess of acetyl chloride was distilled off. The precipitated crystals yield 19.72 g (93%), mp. 66–68°C.

[1.3.] E-3,3,3-tribromo-1-nitroprop-1-en

A mixture of 19.72 g (0.051 mol) of compound (1.2) in 40 ml of benzene and 6 g (0.057 mol) of sodium carbonate was refluxed during 4.5 h. The resulting precipitate was filtered off and the left residue was washed with diethyl ether (4 x 25 ml). Resulting extracts was dried over $CaCl_2$, and distilled off to remove solvent. Dark-orange oil appeared, which was a mixture of isomers. E-3,3,3-Tribromo-1-nitropropene was isolated by column chromatography (eluting with n-hexane:chloroform 0.622:0.375). Obtained yellowish crystals 12.68 g (65%), mp. 48–49°C

[1.4.] Z-C-aryl-N-phenylnitrones. General procedure

A solution of N-phenylhydroxylamine (0.11mol) and appropriate aldehyde (0.10mol) in the ethanol (50 mL) was mixed at room temperature for 24h. The products was

filtered off, washed by small amount of the diethyl ether and recrystallized from the mixture ethanol/hexane.

*[1.5.] Cycloaddition between Z-C-aryl-N-phenylnitrones (**1a-g**) and TBMN (**2**). General Procedure*

A solution of 3,3,3-bromo-1-nitroprop-1-ene (0.02 mol) and appropriate nitrone (0.01 mol) in dry benzene (25 mL) was mixed at room temperature for 24 h. The post-reaction mixture was filtered, and the solvent was evaporated *in vacuo*. The isolation of the reaction products from the post-reaction mixtures was performed via crystallization from ethanol. Pure products were identified on the basis of HR-MS, IR and NMR spectral data.

[2] Physical characteristics of adducts

3,4-cis-4,5-trans-2-phenyl-3-(4-methoxyphenyl)-4-nitro-5-tribromomethylisoxazolidine ***4a***

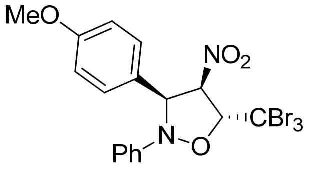

$C_{17}H_{15}N_2O_4Br_3$ Pale yellow crystal needles; m.p. 121-121,3°C from ethanol.

IR: 1552, 1368 (NO_2); 1251, 1085(CH_3-O-Ar); 1175 (-C-N-); 971 (-C-O-); 926 (-N-O-); 665 (-C-Br)

^{1}HNMR (400 MHz, $CDCl_3$): δ 7.37-7.31 (m, 2H), 7.27-7.19 (m, 4H), 7.17-7.11 (m, 1H), 6.91-6.85 (m, 2H), 5.76 (d, J_{H-H} = 4.7 Hz, 1H), 5.54 (dd, J_{H-H} = 8.3 Hz, J_{H-H} = 4.7 Hz, 1H), 4.96 (d, J_{H-H} = 8.3 Hz, 1H), 3.82 (s, 3H) ppm.

^{13}CNMR (400 MHz, $CDCl_3$): δ 160.6, 146.8, 129.5, 128.8, 126.2, 123.1, 120.9, 114.5, 95.6, 89.4, 74.6, 55.2, 37.6 ppm.

HRMS (+APCI): *m/z* calcd for $C_{17}H_{16}N_2O_4Br_3$: 548.8660 $[M+H]^+$; found 548.8668.

3,4-cis-4,5-trans-2-phenyl-3-(4-methylphenyl)-4-nitro-5-tribromomethylisoxazolidine ***4b***

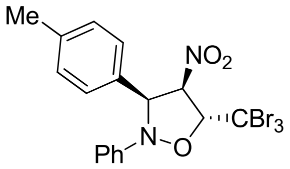

$C_{17}H_{15}N_2O_3Br_3$ White crystal powder; m.p. 130-130,4°C from ethanol.
IR: 1555, 1368 (NO_2); 1182 (-C-N-); 972 (-C-O-); 929 (-N-O-); 668 (-C-Br)

^{1}HNMR (400 MHz, $CDCl_3$): δ 7.33-7.21 (m, 6H), 7.19-7.12 (m, 3H), 5.78 (d, J_{H-H} = 4.7 Hz, 1H), 5.56 (dd, J_{H-H} = 8.4 Hz, J_{H-H} = 4.7 Hz, 1H), 5.00 (d, J_{H-H} = 8.3 Hz, 1H), 2.35 (s, 3H) ppm.

^{13}CNMR (400 MHz, $CDCl_3$): δ 147.0, 140.0, 129.9, 129.0, 128.5, 128.2, 126.4, 121.1, 95.9, 89.6, 74.9, 37.7, 21.5 ppm.

HRMS (+APCI): *m/z* calcd for $C_{17}H_{16}N_2O_3Br_3$: 532.8711 $[M+H]^+$; found 532.8717.

3,4-cis-4,5-trans-2,3-diphenyl-4-nitro-5-tribromomethylisoxazolidine ***4c***

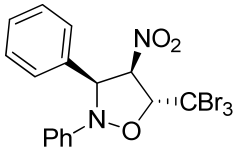

$C_{16}H_{13}N_2O_3Br_3$ Pale yellow crystal needles; m.p. 148,9-149,1°C from ethanol. IR: 1554, 1366 (NO_2); 1162 (-C-N-); 986 (-C-O-); 920 (-N-O-); 662 (-C-Br)

^{1}HNMR (600 MHz, $CDCl_3$): δ 7.49-7.43 (m, 2H), 7.41-7.36 (m, 3H), 7.30-7.22 (m, 4H), 7.18-7.13 (m, 1H), 5.80 (d, J_{H-H} = 4.8 Hz, 1H), 5.60 (dd, J_{H-H} = 8.4 Hz, J_{H-H} = 4.7 Hz, 1H), 5.05 (d, J_{H-H} = 8.3 Hz, 1H) ppm.

^{13}CNMR (151 MHz, $CDCl_3$): δ 146.7, 131.5, 129.9, 129.1, 128.9, 128.3, 126.3, 121.0, 95.8, 89.5, 74.8, 37.5 ppm.

HRMS (+APCI): *m/z* calcd for $C_{16}H_{14}N_2O_3Br_3$: 518.8555 $[M+H]^+$; found 518.8565.

3,4-cis-4,5-trans-2-phenyl-3-(4-fluorphenyl)-4-nitro-5-tribromomethylisoxazolidine ***4d***

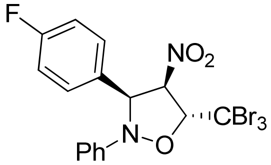

$C_{16}H_{12}N_2O_3Br_3F$ Pale yellow crystal plates; m.p. 118-121°C from ethanol.
IR: 1560, 1366 (NO_2); 1156 (-C-N-); 969 (-C-O-); 929 (-N-O-); 640 (-C-Br)

^{1}HNMR (400 MHz, $CDCl_3$): δ 7.45-7.39 (m, 2H), 7.31-7.25 (m, 2H), 7.23-7.15 (m, 3H), 7.09-7.03 (m, 2H), 5.77 (d, J_{H-H} = 4.7 Hz, 1H), 5.57 (dd, J_{H-H} = 8.4 Hz, J_{H-H} = 4.7 Hz, 1H), 5.01 (d, J_{H-H} = 8.2 Hz, 1H) ppm.

^{13}CNMR (400 MHz, $CDCl_3$): δ 146.6, 130.3 (d, J_{C-F} = 8.5 Hz), 129.2, 127.4 3 (d, J_{C-F} = 8.5 Hz), 126.8, 121.3, 116.6, 116.3, 95.7, 89.6, 74.2, 37.5 ppm.

^{19}FNMR (400 MHz, $CDCl_3$): δ -109.92 ppm.

HRMS (+APCI): *m/z* calcd for $C_{16}H_{13}N_2O_3Br_3F_1$: 536.8461 $[M+H]^+$; found 536.8460.

3,4-cis-4,5-trans-2-phenyl-3-(4-chlorophenyl)-4-nitro-5-tribromomethylisoxazolidine ***4e***

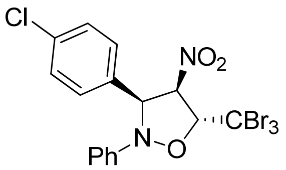

$C_{16}H_{12}N_2O_3Br_3Cl$ Pale yellow crystal needles; m.p. 152,5-153°C from ethanol. IR: 1556, 1368 (NO_2); 1177 (-C-N-); 968 (-C-O-); 931(-N-O-); 635 (-C-Br)

^{1}HNMR (400 MHz, $CDCl_3$): δ 7.40-7.33 (m, 4H), 7.31-7.25 (m, 2H), 7.24-7.15 (m, 3H), 5.77 (d, J_{H-H} = 4.8 Hz, 1H), 5.58 (dd, J_{H-H} = 8.4 Hz, J_{H-H} = 4.8 Hz, 1H), 5.01 (d, J_{H-H} = 8.3 Hz, 1H) ppm.

^{13}CNMR (400 MHz, $CDCl_3$): δ 146.5, 136.1, 130.1, 129.7, 129.6, 129.2, 126.8, 121.3, 95.7, 89.6, 74.1, 37.4 ppm.

HRMS (+APCI): *m/z* calcd for $C_{16}H_{13}N_2O_3Br_3Cl_1$: 552.8167 $[M+H]^+$; found 552.8165.

3,4-cis-4,5-trans-2-phenyl-3-(4-bromophenyl)-4-nitro-5-tribromomethylisoxazolidine ***4f***

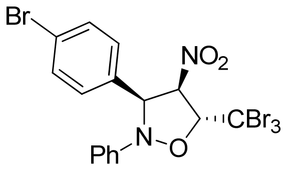

$C_{16}H_{12}N_2O_3Br_4$ White crystal powder; m.p. 126-128°C from ethanol.
IR: 1552, 1367 (NO_2); 1181 (-C-N-); 977 (-C-O-); 929 (-N-O-); 664 (-C-Br)

^{1}HNMR (400 MHz, $CDCl_3$): δ 7.54-7.47 (m, 2H), 7.33-7.25 (m, 4H), 7.22-7.14 (m, 3H), 5.75 (d, J_{H-H} = 4.7 Hz, 1H), 5.57 (dd, J_{H-H} = 8.3 Hz, J_{H-H} = 4.7 Hz, 1H), 4.99 (d, J_{H-H} = 8.2 Hz, 1H) ppm.

^{13}CNMR (400 MHz, $CDCl_3$): δ 146.3, 132.3, 130.5, 129.8, 129.0, 126.6, 124.2, 121.1, 95.4, 89.4, 74.0, 37.2 ppm.

HRMS (+APCI): *m/z* calcd for $C_{16}H_{13}N_2O_3Br_4$: 596.7660 $[M+H]^+$; found 596.7672

3,4-cis-4,5-trans-2-phenyl-3-(4-carbomethoxyphenyl)-4-nitro-5-tribromomethylisoxazolidine ***4g***

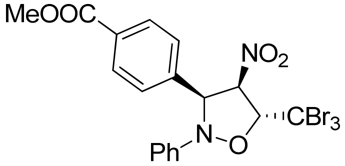

$C_{17}H_{15}N_2O_5Br_3$ Pale orange crystal powder; m.p. 133,7-134,2°C from ethanol. IR: 1706 (C=O); 1561, 1364 (NO_2); 1191 (-C-N-); 991 (-C-O-); 921 (-N-O-); 661 (-C-Br)

^{1}HNMR (400 MHz, $CDCl_3$): δ 7.94-7.86 (m, 1H), 7.83-7.77 (m, 1H), 7.49-7.26 (m, 7H), 5.80 (d, J_{H-H} = 4.7 Hz, 1H), 5.61 (dd, J_{H-H} = 8.3 Hz, J_{H-H} = 4.5 Hz, 1H), 5.09 (d, J_{H-H} = 8.3 Hz, 1H), 3.88 (s, 3H) ppm.

^{13}CNMR (400 MHz, $CDCl_3$): δ 166.4, 146.7, 130.1, 129.1, 129.0, 128.2, 127.1, 125.3, 121.7, 95.5, 89.5, 74.4, 52.3, 37.3 ppm.

HRMS (+APCI): *m*/*z* calcd for $C_{18}H_{16}N_2O_5Br_3$: 576.8609 $[M+H]^+$; found 576.8616.

[3] NMR spectra of adducts

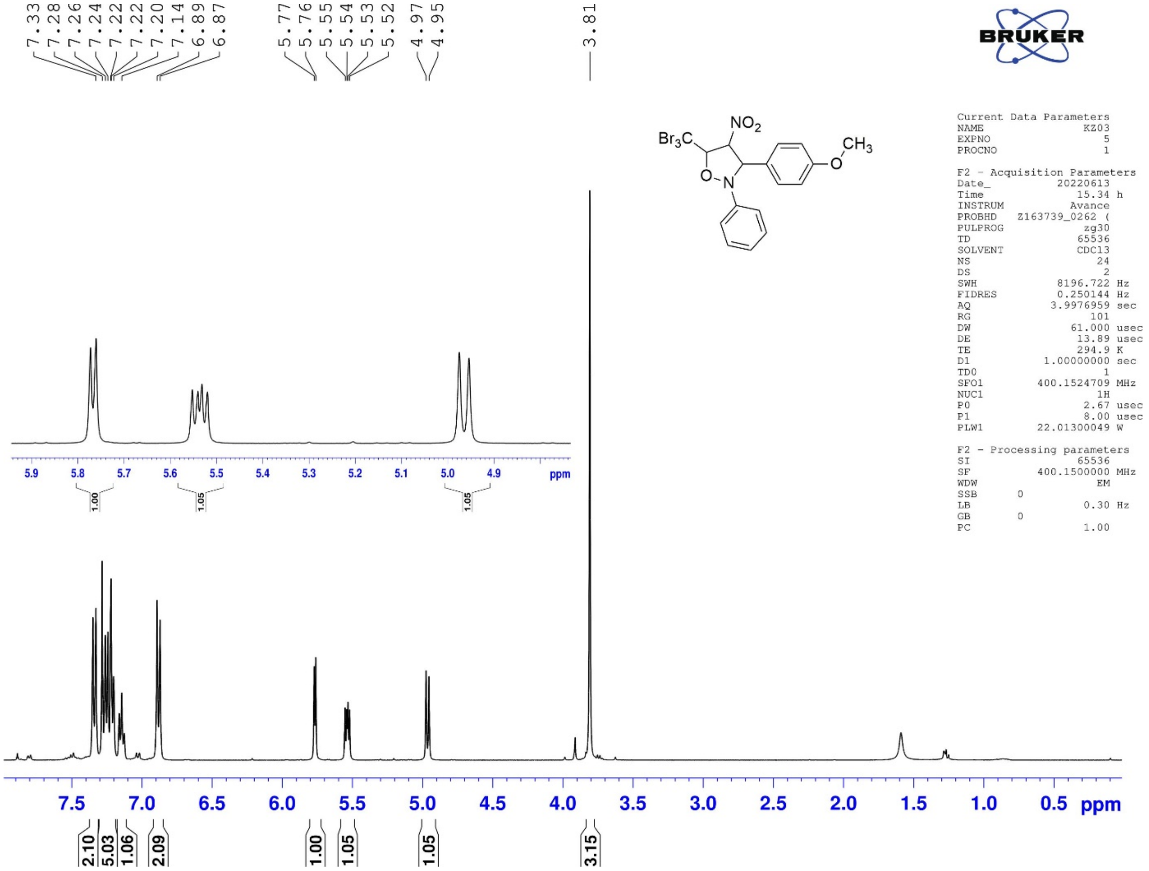
7.33
7.28
7.26
7.24
7.22
7.22
7.20
7.14
6.89
6.87
5.77
5.76
5.55
5.54
5.53
5.52
4.97
4.95
3.81
BRUKER
NO_2
Br_3C
CH_3
O
N
Current Data Parameters
NAME KZ03
EXPNO 5
PROCNO 1
F2 - Acquisition Parameters
Date_ 20220613
Time 15.34 h
INSTRUM Avance
PROBHD Z163739_0262 (
PULPROG zg30
TD 65536
SOLVENT CDCl3
NS 24
DS 2
SWH 8196.722 Hz
FIDRES 0.250144 Hz
AQ 3.9976959 sec
RG 101
DW 61.000 usec
DE 13.89 usec
TE 294.9 K
D1 1.00000000 sec
TD0 1
SFO1 400.1524709 MHz
NUC1 1H
P0 2.67 usec
P1 8.00 usec
PLW1 22.01300049 W
F2 - Processing parameters
SI 65536
SF 400.1500000 MHz
WDW EM
SSB 0
LB 0.30 Hz
GB 0
PC 1.00
ppm
1.00
1.05
1.05
2.10
5.03
1.06
2.09
1.00
1.05
1.05
3.15

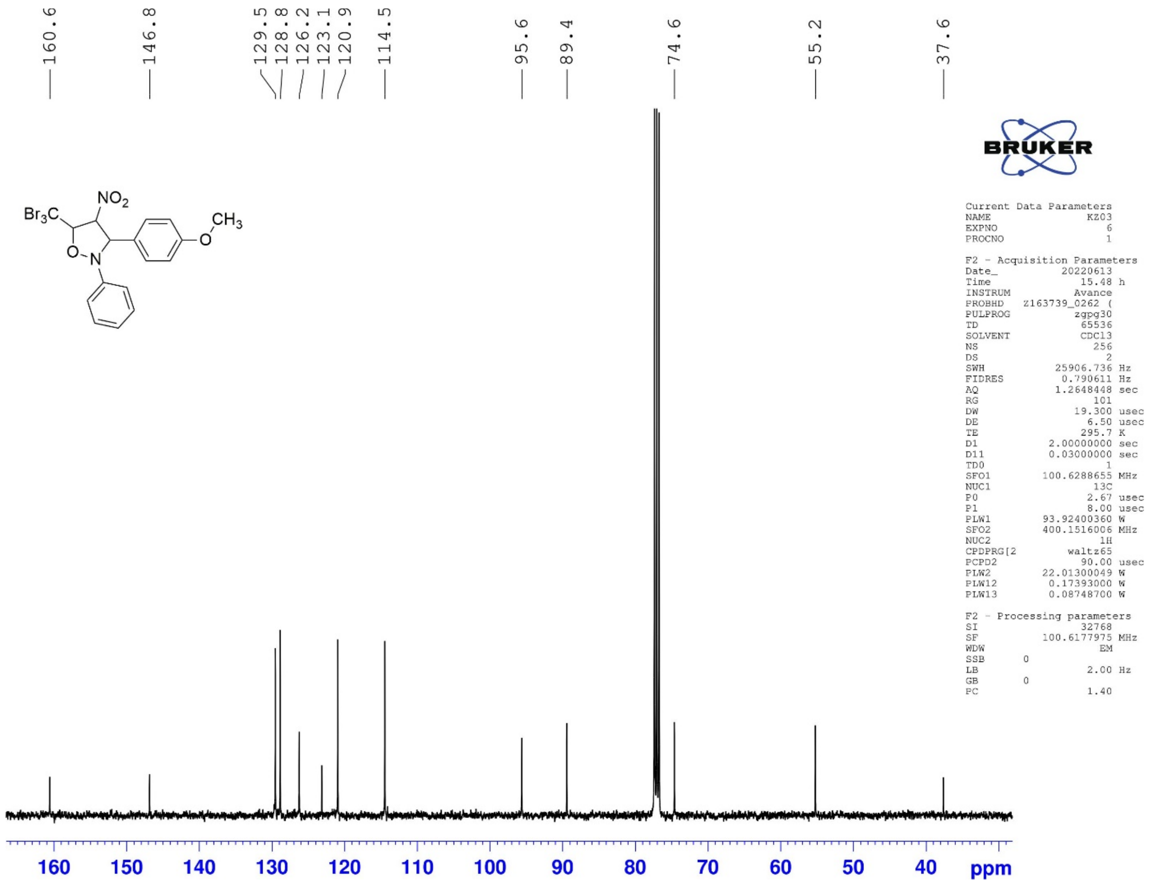
160.6
146.8
129.5
128.8
126.2
123.1
120.9
114.5
95.6
89.4
74.6
55.2
37.6
Br3C
NO2
CH3
O
O
N
BRUKER
Current Data Parameters
NAME KZ03
EXPNO 6
PROCNO 1
F2 - Acquisition Parameters
Date_ 20220613
Time 15.48 h
INSTRUM Avance
PROBHD Z163739_0262 (
PULPROG zgpg30
TD 65536
SOLVENT CDCl3
NS 256
DS 2
SWH 25906.736 Hz
FIDRES 0.790611 Hz
AQ 1.2648448 sec
RG 101
DW 19.300 usec
DE 6.50 usec
TE 295.7 K
D1 2.00000000 sec
D11 0.03000000 sec
TD0 1
SFO1 100.6288655 MHz
NUC1 13C
P0 2.67 usec
P1 8.00 usec
PLW1 93.92400360 W
SFO2 400.1516006 MHz
NUC2 1H
CPDPRG[2 waltz65
PCPD2 90.00 usec
PLW2 22.01300049 W
PLW12 0.17393000 W
PLW13 0.08748700 W
F2 - Processing parameters
SI 32768
SF 100.6177975 MHz
WDW EM
SSB 0
LB 2.00 Hz
GB 0
PC 1.40
160 150 140 130 120 110 100 90 80 70 60 50 40 ppm

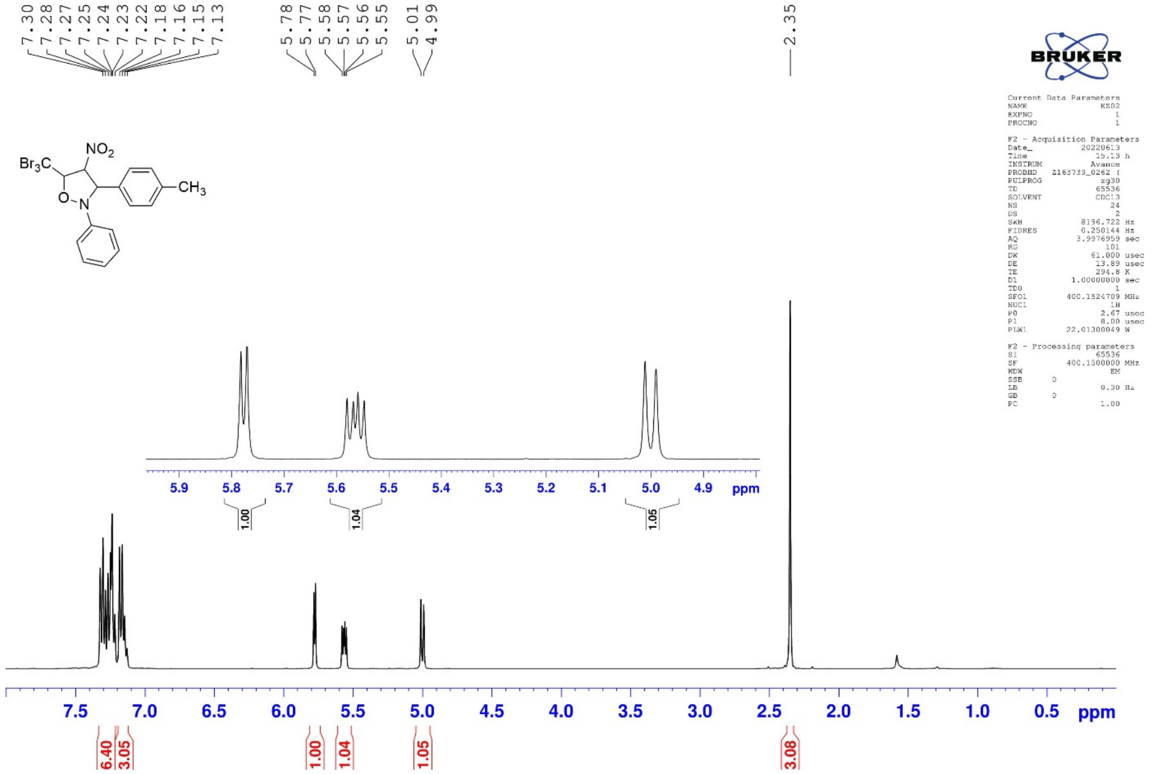
7.30
7.28
7.27
7.25
7.24
7.23
7.22
7.18
7.16
7.15
7.13
5.78
5.77
5.58
5.57
5.56
5.55
5.01
4.99
2.35
Br3C
NO2
CH3
O
N
BRUKER
5.9 5.8 5.7 5.6 5.5 5.4 5.3 5.2 5.1 5.0 4.9 ppm
1.00
1.04
1.05
7.5 7.0 6.5 6.0 5.5 5.0 4.5 4.0 3.5 3.0 2.5 2.0 1.5 1.0 0.5 ppm
6.40
3.05
1.00
1.04
1.05
3.08

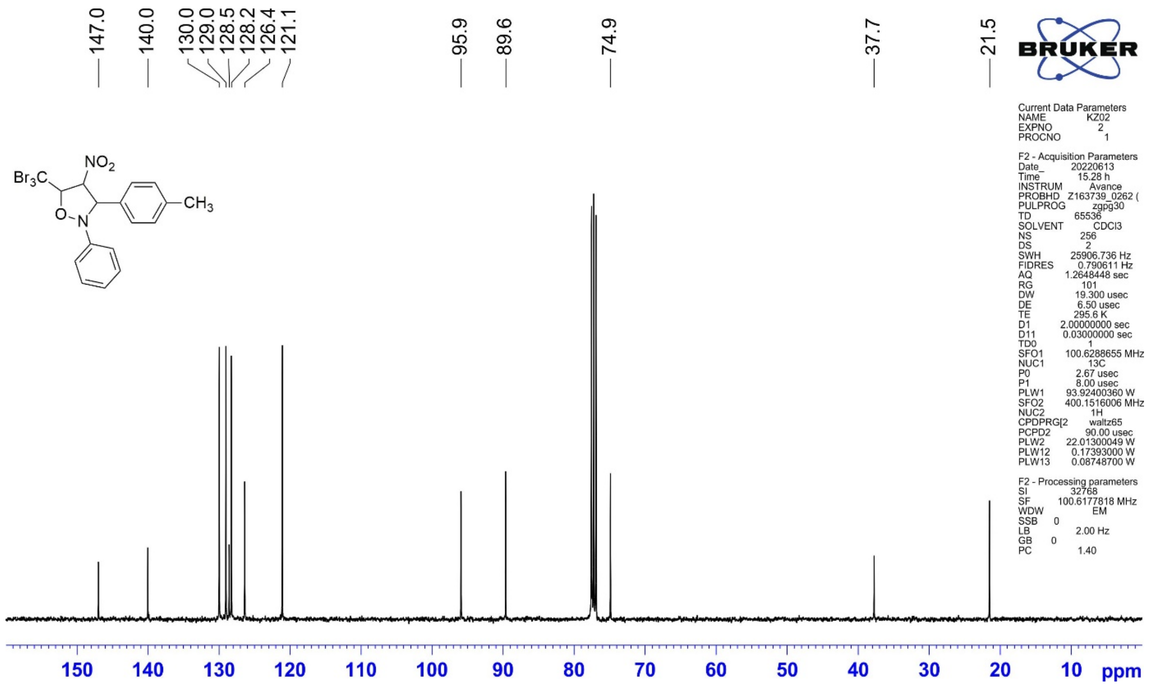

147.0
140.0
130.0
129.0
128.5
128.2
126.4
121.1
95.9
89.6
74.9
37.7
21.5
Br_3C
NO_2
CH_3
O
N
Current Data Parameters
NAME KZ02
EXPNO 2
PROCNO 1
F2 - Acquisition Parameters
Date_ 20220613
Time 15.28 h
INSTRUM Avance
PROBHD Z163739_0262 (
PULPROG zgpg30
TD 65536
SOLVENT CDCl3
NS 256
DS 2
SWH 25906.736 Hz
FIDRES 0.790611 Hz
AQ 1.2648448 sec
RG 101
DW 19.300 usec
DE 6.50 usec
TE 295.6 K
D1 2.00000000 sec
D11 0.03000000 sec
TD0 1
SFO1 100.6288655 MHz
NUC1 13C
P0 2.67 usec
P1 8.00 usec
PLW1 93.92400360 W
SFO2 400.1516006 MHz
NUC2 1H
CPDPRG[2 waltz65
PCPD2 90.00 usec
PLW2 22.01300049 W
PLW12 0.17393000 W
PLW13 0.08748700 W
F2 - Processing parameters
SI 32768
SF 100.6177818 MHz
WDW EM
SSB 0
LB 2.00 Hz
GB 0
PC 1.40
150 140 130 120 110 100 90 80 70 60 50 40 30 20 10 ppm

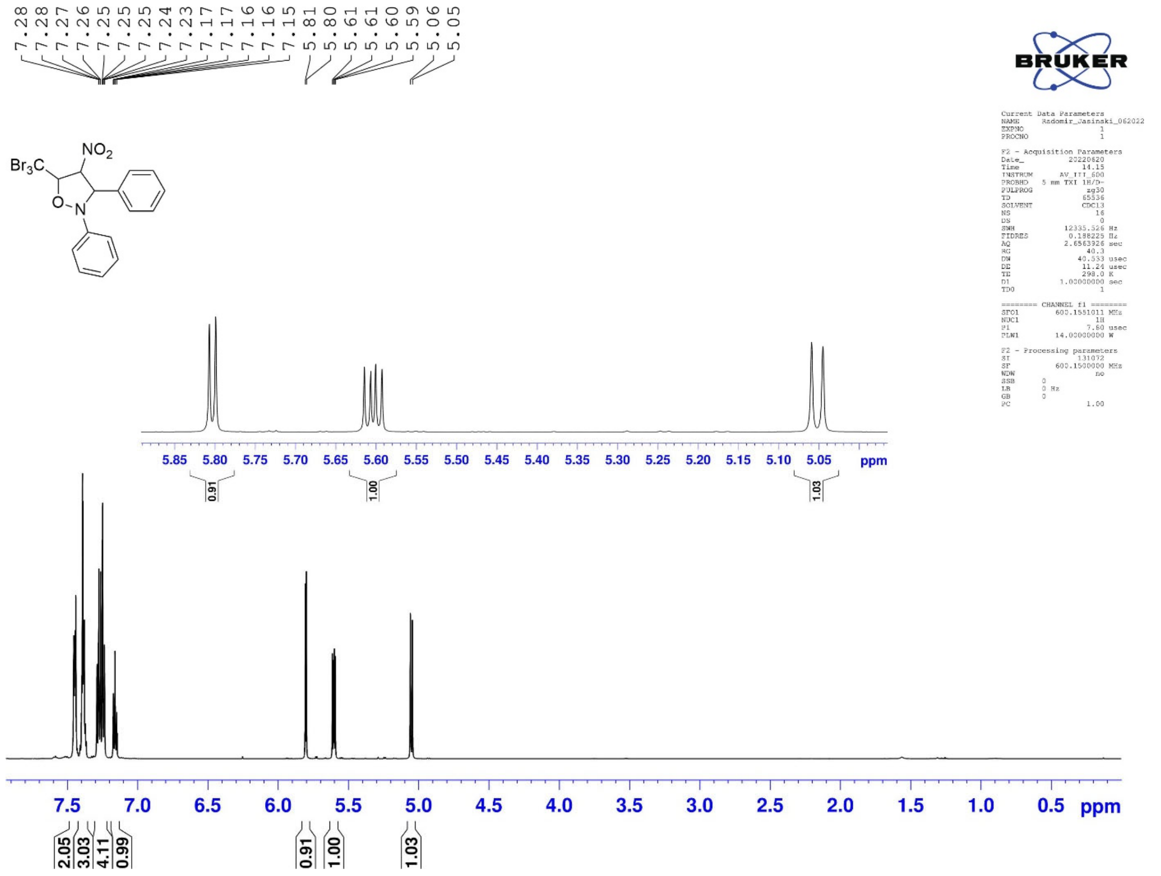

7.28
7.28
7.27
7.26
7.25
7.25
7.25
7.24
7.23
7.17
7.17
7.16
7.16
7.15
5.81
5.80
5.61
5.61
5.60
5.59
5.06
5.05
Br_3C
NO_2
O
N
Current Data Parameters
NAME Radomir_Jasinski_062022
EXPNO 1
PROCNO 1
F2 - Acquisition Parameters
Date_ 20220620
Time 14.15
INSTRUM AV_III_600
PROBHD 5 mm TXI 1H/D-
PULPROG zg30
TD 65536
SOLVENT CDCl3
NS 16
DS 0
SWH 12335.526 Hz
FIDRES 0.188225 Hz
AQ 2.6563926 sec
RG 40.3
DW 40.533 usec
DE 11.24 usec
TE 298.0 K
D1 1.00000000 sec
TD0 1
======== CHANNEL f1 ========
SFO1 600.1551011 MHz
NUC1 1H
P1 7.60 usec
PLW1 14.00000000 W
F2 - Processing parameters
SI 131072
SF 600.1500000 MHz
WDW no
SSB 0
LB 0 Hz
GB 0
PC 1.00
5.85 5.80 5.75 5.70 5.65 5.60 5.55 5.50 5.45 5.40 5.35 5.30 5.25 5.20 5.15 5.10 5.05 ppm
0.91
1.00
1.03
7.5 7.0 6.5 6.0 5.5 5.0 4.5 4.0 3.5 3.0 2.5 2.0 1.5 1.0 0.5 ppm
2.05
3.03
4.11
0.99
0.91
1.00
1.03

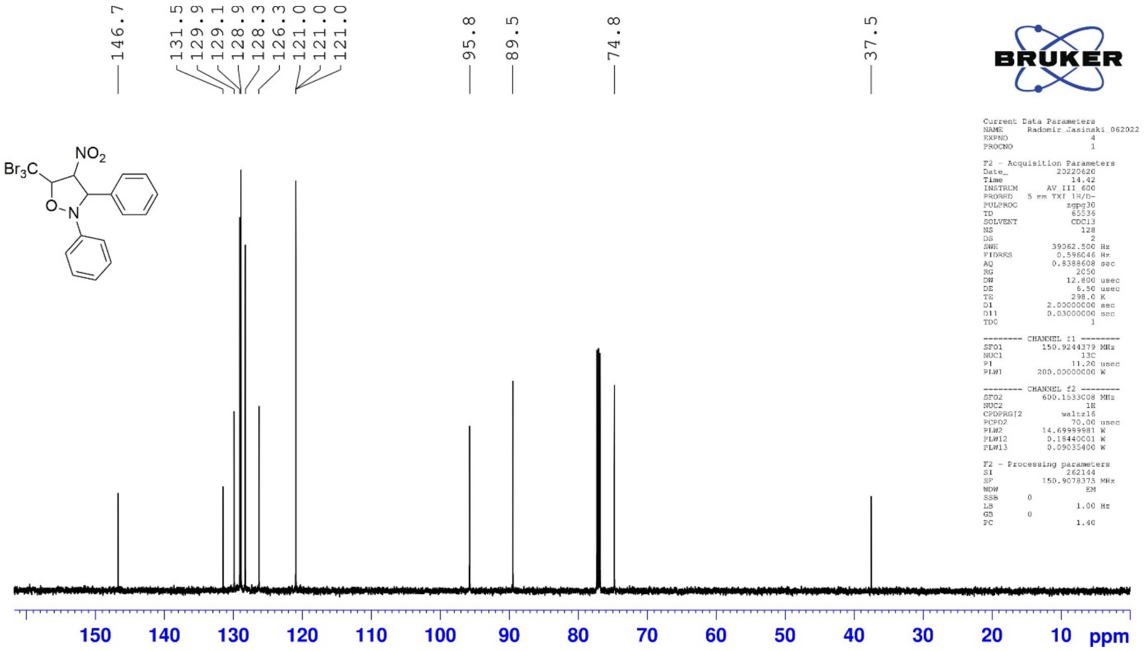

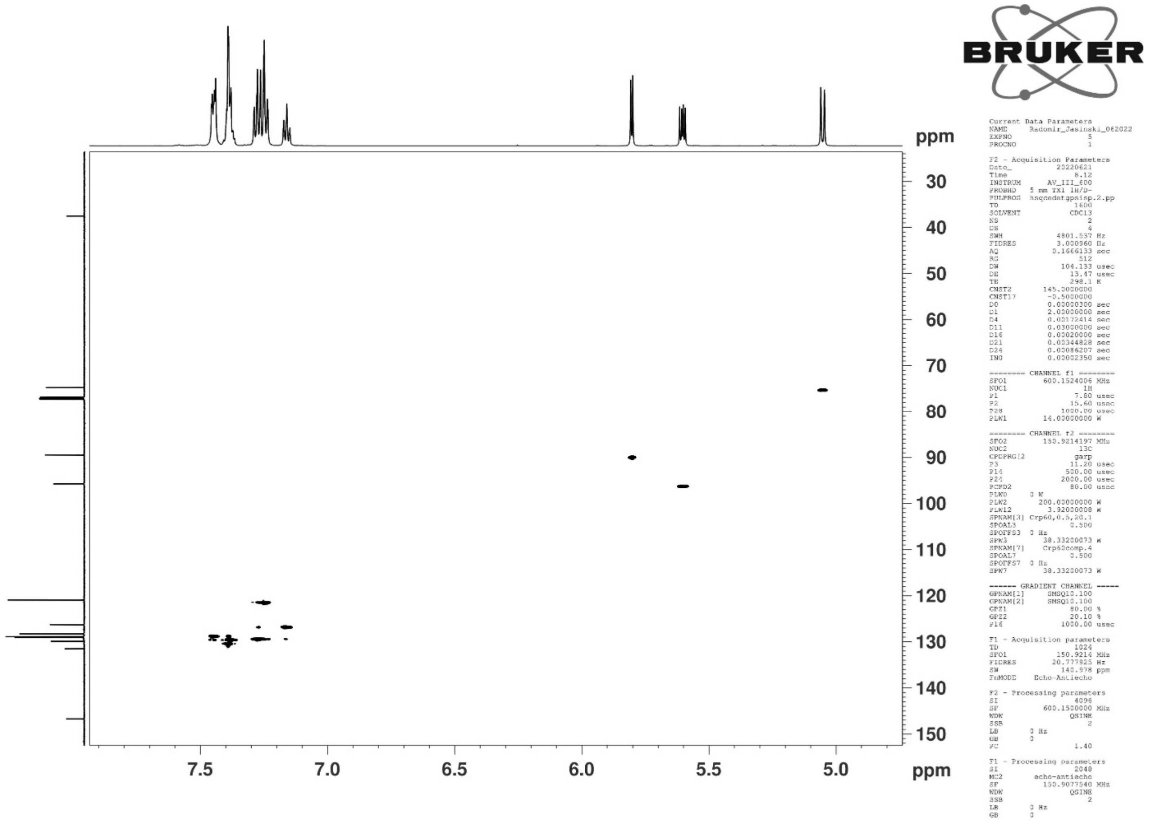

HSQC spectrum of *3,4-cis-4,5-trans-2,3-diphenyl-4-nitro-5-tribromomethylisoxazolidine* ***4c***.

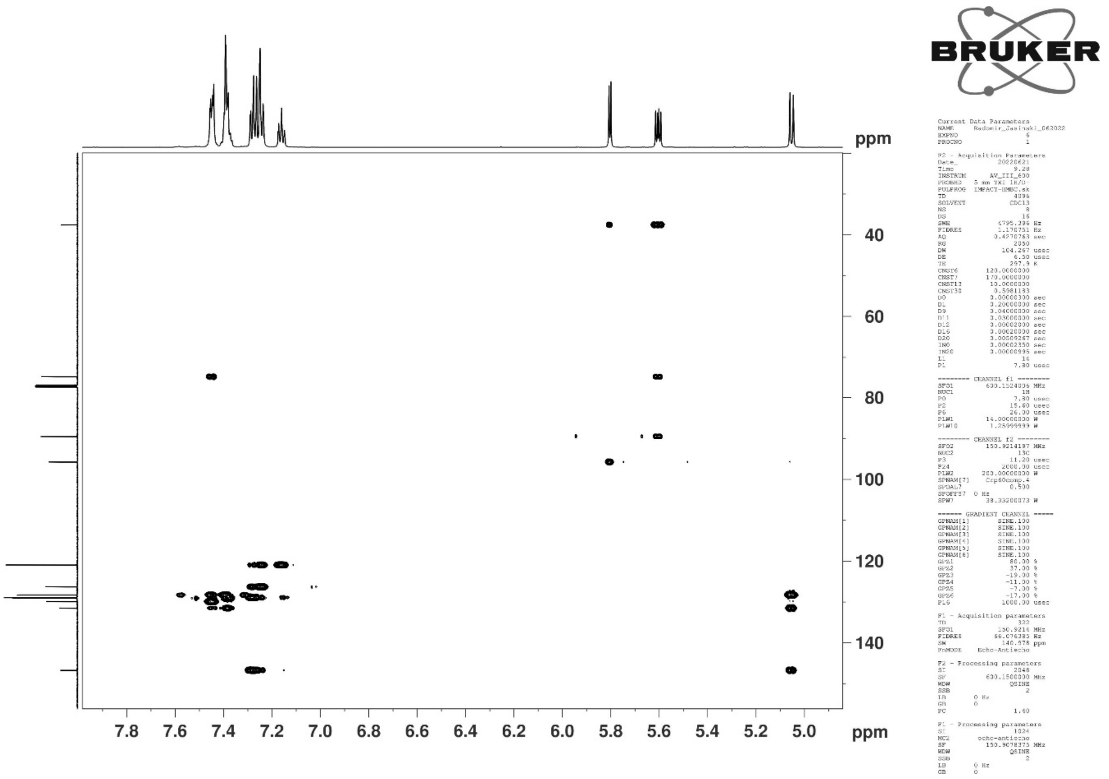

^1H-^{13}C HMBC spectrum of *3,4-cis-4,5-trans-2,3-diphenyl-4-nitro-5-tribromomethylisoxazolidine* ***4c***.

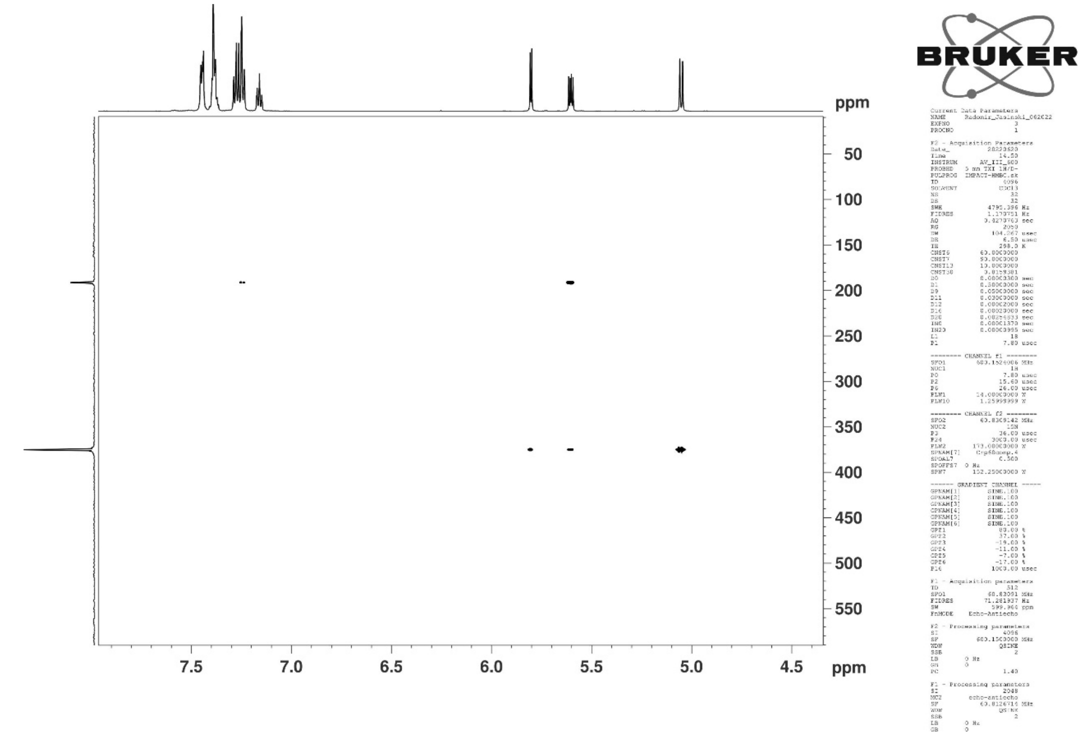

^{1}H-^{15}N HMBC spectrum of *3,4-cis-4,5-trans-2,3-diphenyl-4-nitro-5-tribromomethylisoxazolidine* **4c**.

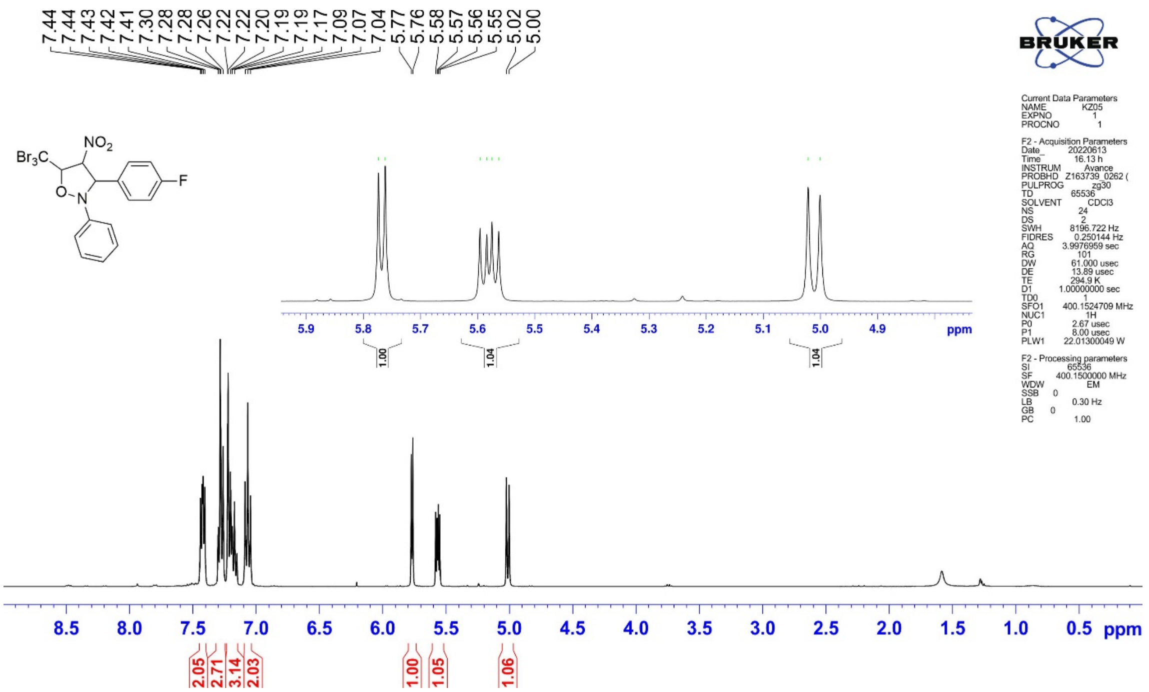

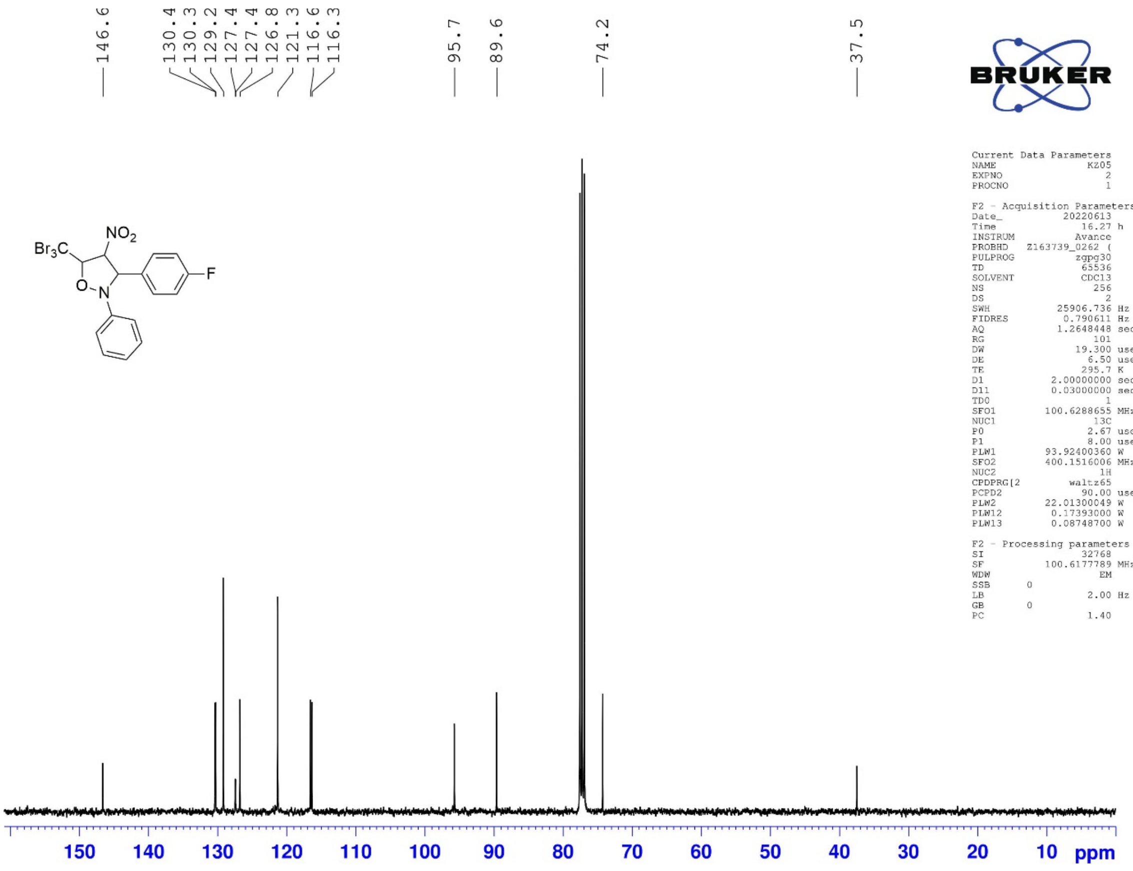


```
Current Data Parameters
NAME                 KZ05
EXPNO                   2
PROCNO                  1

F2 - Acquisition Parameters
Date_            20220613
Time                16.27 h
INSTRUM            Avance
PROBHD    Z163739_0262 (
PULPROG           zgpg30
TD                 65536
SOLVENT            CDCl3
NS                    256
DS                      2
SWH             25906.736 Hz
FIDRES           0.790611 Hz
AQ              1.2648448 sec
RG                    101
DW                 19.300 usec
DE                   6.50 usec
TE                  295.7 K
D1             2.00000000 sec
D11            0.03000000 sec
TD0                     1
SFO1          100.6288655 MHz
NUC1                  13C
P0                   2.67 usec
P1                   8.00 usec
PLW1          93.92400360 W
SFO2          400.1516006 MHz
NUC2                   1H
CPDPRG[2          waltz65
PCPD2               90.00 usec
PLW2          22.01300049 W
PLW12          0.17393000 W
PLW13          0.08748700 W

F2 - Processing parameters
SI                  32768
SF            100.6177789 MHz
WDW                    EM
SSB      0
LB                   2.00 Hz
GB       0
PC                   1.40
```

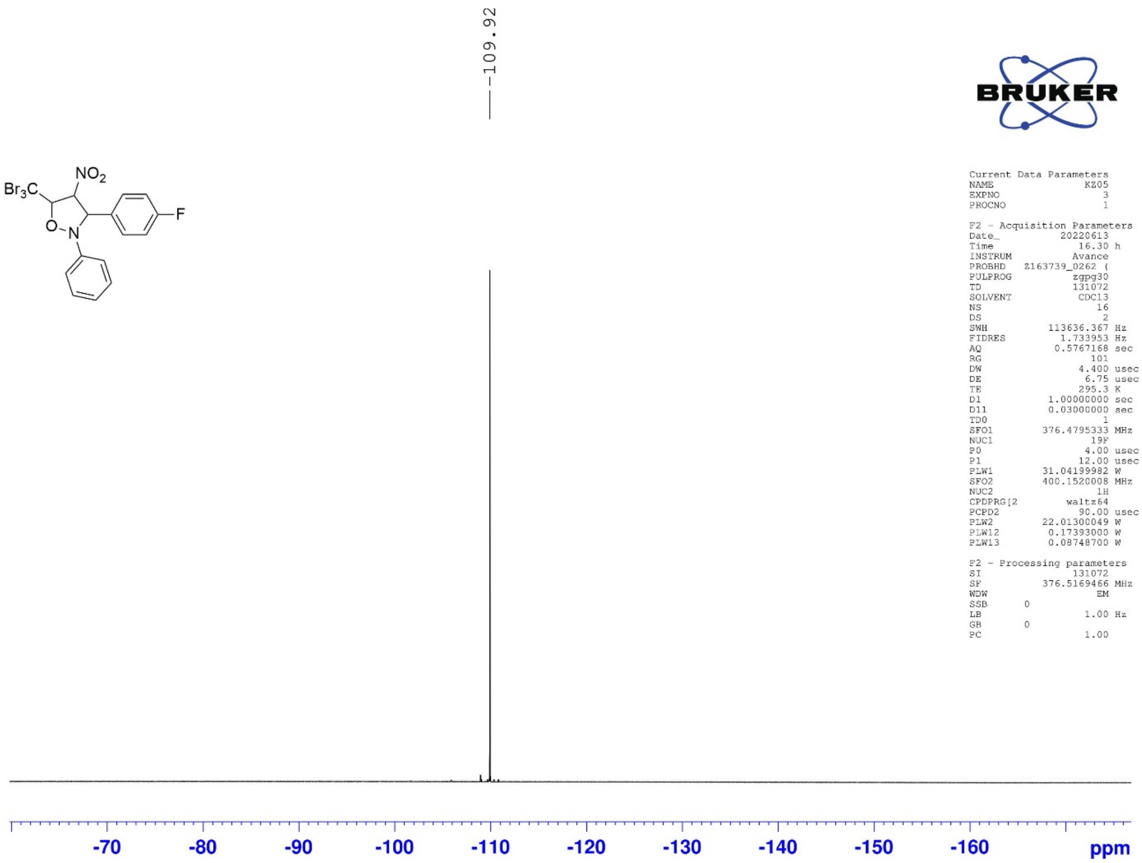
-109.92
Br_3C
NO_2
O
N
F
BRUKER
Current Data Parameters
NAME KZ05
EXPNO 3
PROCNO 1
F2 - Acquisition Parameters
Date_ 20220613
Time 16.30 h
INSTRUM Avance
PROBHD Z163739_0262 (
PULPROG zgpg30
TD 131072
SOLVENT CDCl3
NS 16
DS 2
SWH 113636.367 Hz
FIDRES 1.733953 Hz
AQ 0.5767168 sec
RG 101
DW 4.400 usec
DE 6.75 usec
TE 295.3 K
D1 1.00000000 sec
D11 0.03000000 sec
TD0 1
SFO1 376.4795333 MHz
NUC1 19F
P0 4.00 usec
P1 12.00 usec
PLW1 31.04199982 W
SFO2 400.1520008 MHz
NUC2 1H
CPDPRG[2 waltz64
PCPD2 90.00 usec
PLW2 22.01300049 W
PLW12 0.17393000 W
PLW13 0.08748700 W
F2 - Processing parameters
SI 131072
SF 376.5169466 MHz
WDW EM
SSB 0
LB 1.00 Hz
GB 0
PC 1.00
-70 -80 -90 -100 -110 -120 -130 -140 -150 -160 ppm

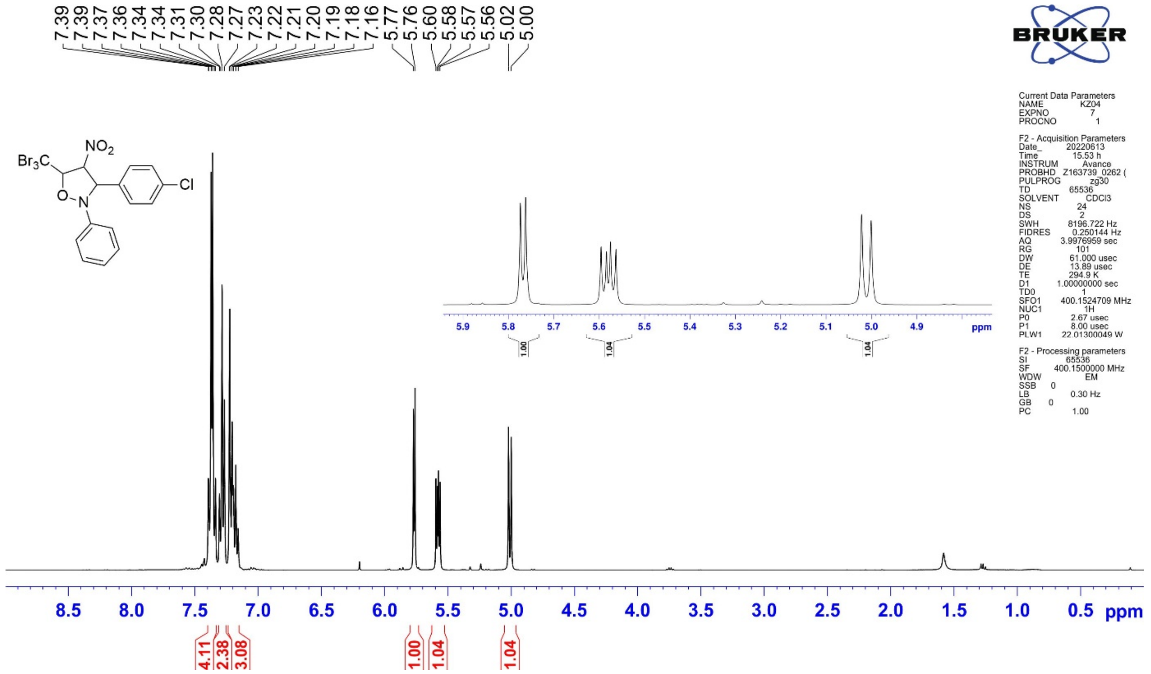
7.39 7.39 7.37 7.36 7.34 7.34 7.31 7.30 7.28 7.27 7.23 7.22 7.21 7.20 7.19 7.18 7.16 5.77 5.76 5.60 5.58 5.57 5.56 5.02 5.00
Br_3C
NO_2
O
N
Cl
BRUKER
Current Data Parameters
NAME KZ04
EXPNO 7
PROCNO 1
F2 - Acquisition Parameters
Date_ 20220613
Time 15.53 h
INSTRUM Avance
PROBHD Z163739_0262 (
PULPROG zg30
TD 65536
SOLVENT CDCl3
NS 24
DS 2
SWH 8196.722 Hz
FIDRES 0.250144 Hz
AQ 3.9976959 sec
RG 101
DW 61.000 usec
DE 13.89 usec
TE 294.9 K
D1 1.00000000 sec
TD0 1
SFO1 400.1524709 MHz
NUC1 1H
P0 2.67 usec
P1 8.00 usec
PLW1 22.01300049 W
F2 - Processing parameters
SI 65536
SF 400.1500000 MHz
WDW EM
SSB 0
LB 0.30 Hz
GB 0
PC 1.00
5.9 5.8 5.7 5.6 5.5 5.4 5.3 5.2 5.1 5.0 4.9 ppm
1.00 1.04 1.04
8.5 8.0 7.5 7.0 6.5 6.0 5.5 5.0 4.5 4.0 3.5 3.0 2.5 2.0 1.5 1.0 0.5 ppm
4.11 2.38 3.08 1.00 1.04 1.04

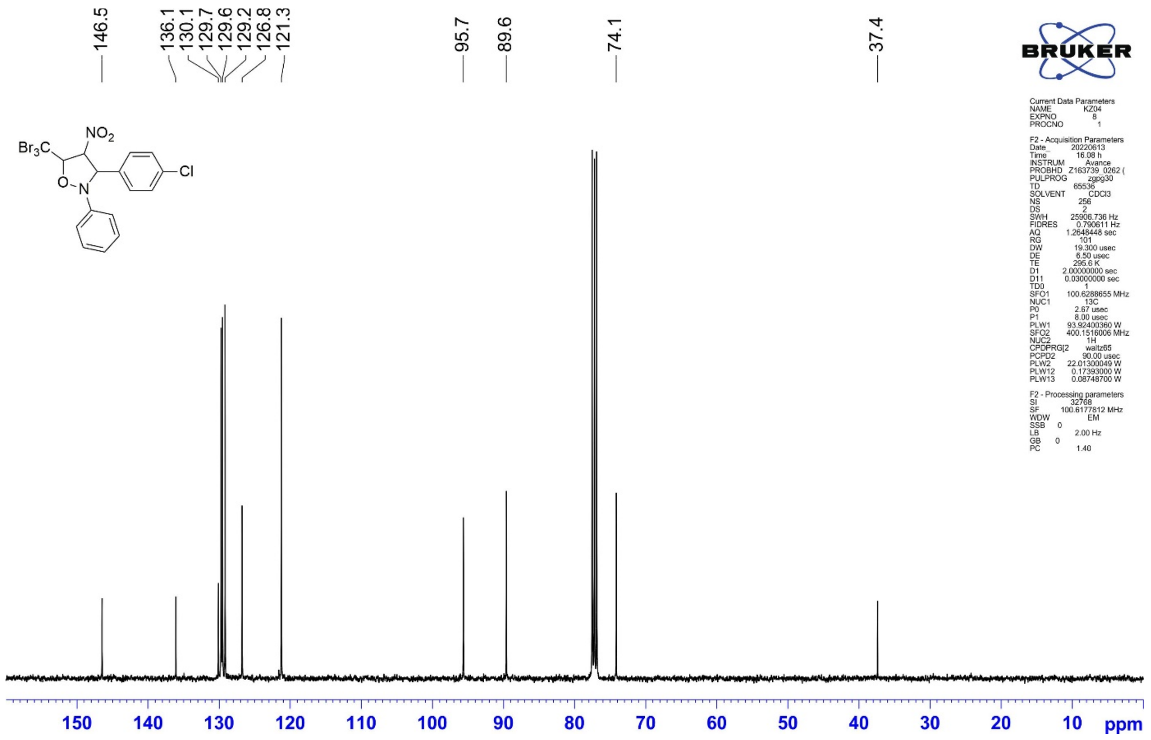
146.5
136.1
130.1
129.7
129.6
129.2
126.8
121.3
95.7
89.6
74.1
37.4
Br_3C
NO_2
Cl
O
N
BRUKER
Current Data Parameters
NAME KZ04
EXPNO 8
PROCNO 1
F2 - Acquisition Parameters
Date_ 20220613
INSTRUM Avance
PULPROG zgpg30
TD 65536
SOLVENT CDCl3
NS 256
DS 2
SFO1 100.6288655 MHz
NUC1 13C
F2 - Processing parameters
SI 32768
SF 100.6177812 MHz
WDW EM
LB 2.00 Hz
PC 1.40
150
140
130
120
110
100
90
80
70
60
50
40
30
20
10
ppm

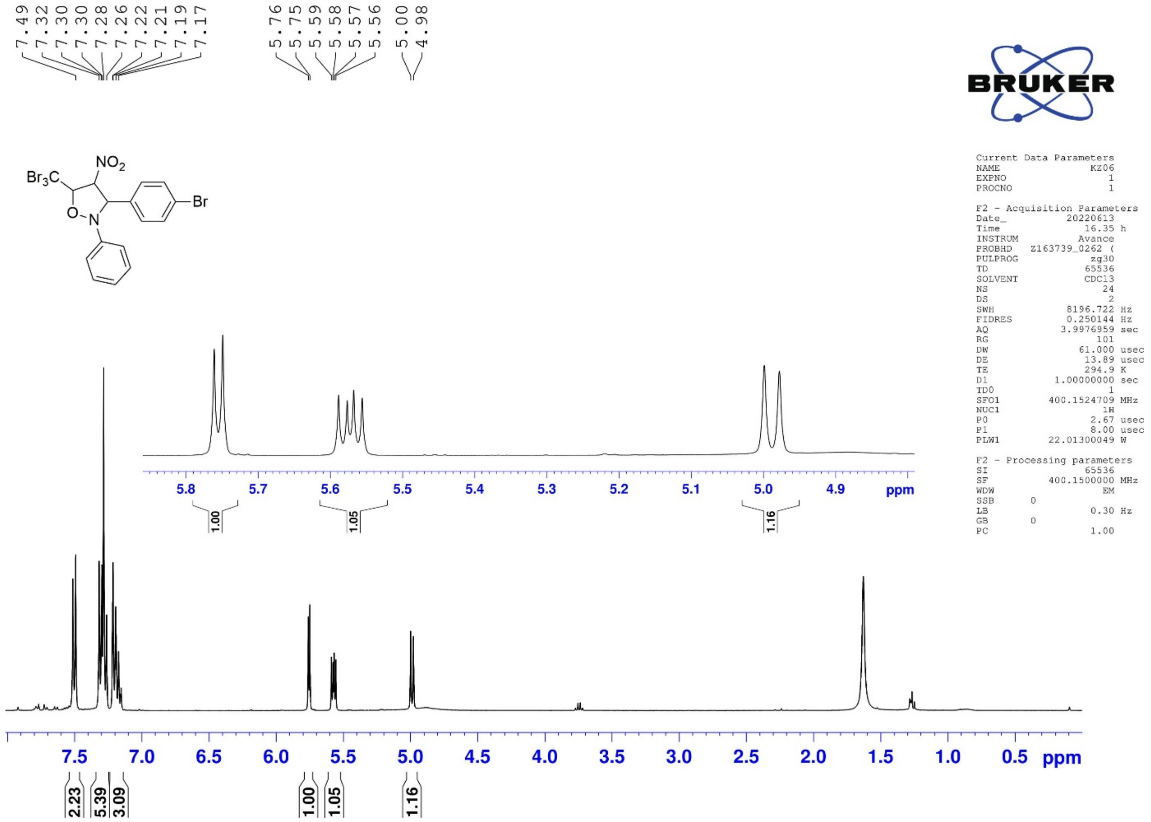
7.49
7.32
7.30
7.30
7.28
7.26
7.22
7.21
7.19
7.17
5.76
5.75
5.59
5.58
5.57
5.56
5.00
4.98
Br_3C
NO_2
Br
O
N
BRUKER
Current Data Parameters
NAME KZ06
EXPNO 1
PROCNO 1
F2 - Acquisition Parameters
Date_ 20220613
Time 16.35 h
INSTRUM Avance
PROBHD Z163739_0262 (
PULPROG zg30
TD 65536
SOLVENT CDCl3
NS 24
DS 2
SWH 8196.722 Hz
FIDRES 0.250144 Hz
AQ 3.9976959 sec
RG 101
DW 61.000 usec
DE 13.89 usec
TE 294.9 K
D1 1.00000000 sec
TD0 1
SFO1 400.1524709 MHz
NUC1 1H
P0 2.67 usec
P1 8.00 usec
PLW1 22.01300049 W
F2 - Processing parameters
SI 65536
SF 400.1500000 MHz
WDW EM
SSB 0
LB 0.30 Hz
GB 0
PC 1.00
5.8
5.7
5.6
5.5
5.4
5.3
5.2
5.1
5.0
4.9
ppm
1.00
1.05
1.16
7.5
7.0
6.5
6.0
5.5
5.0
4.5
4.0
3.5
3.0
2.5
2.0
1.5
1.0
0.5
ppm
2.23
5.39
3.09
1.00
1.05
1.16

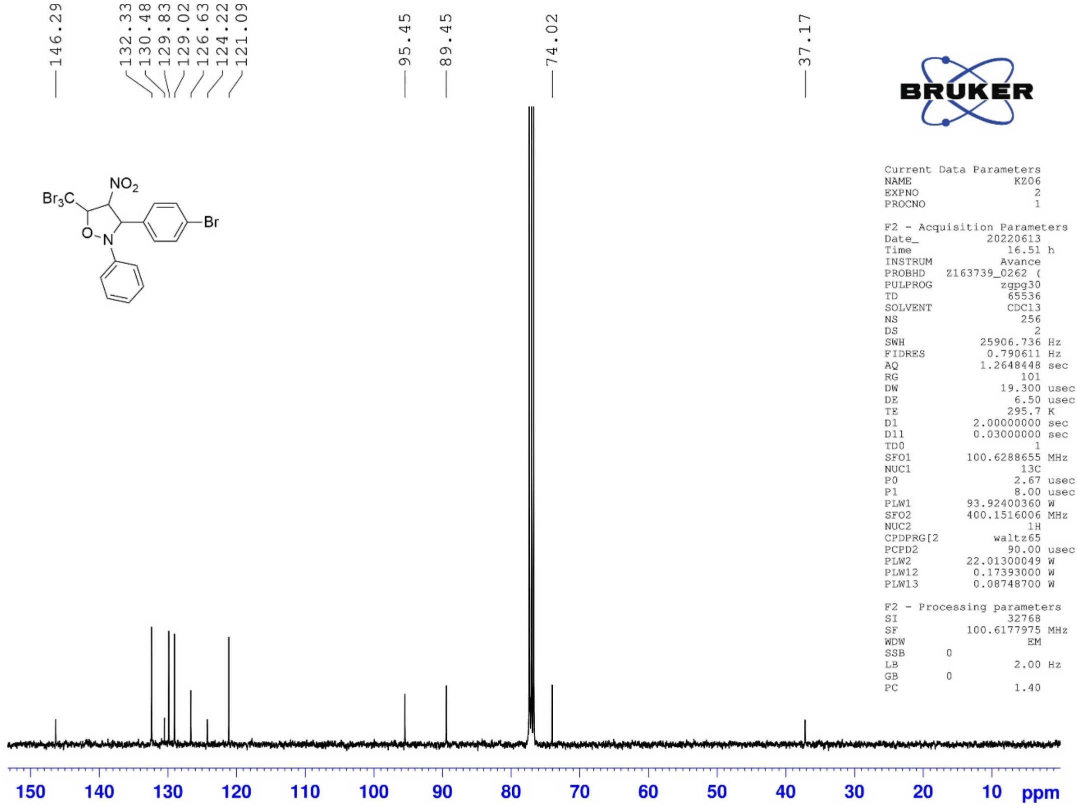
146.29
132.33
130.48
129.83
129.02
126.63
124.22
121.09
95.45
89.45
74.02
37.17
BRUKER
Current Data Parameters
NAME KZ06
EXPNO 2
PROCNO 1
F2 - Acquisition Parameters
Date_ 20220613
Time 16.51 h
INSTRUM Avance
PROBHD Z163739_0262 (
PULPROG zgpg30
TD 65536
SOLVENT CDCl3
NS 256
DS 2
SWH 25906.736 Hz
FIDRES 0.790611 Hz
AQ 1.2648448 sec
RG 101
DW 19.300 usec
DE 6.50 usec
TE 295.7 K
D1 2.00000000 sec
D11 0.03000000 sec
TD0 1
SFO1 100.6288655 MHz
NUC1 13C
P0 2.67 usec
P1 8.00 usec
PLW1 93.92400360 W
SFO2 400.1516006 MHz
NUC2 1H
CPDPRG[2 waltz65
PCPD2 90.00 usec
PLW2 22.01300049 W
PLW12 0.17393000 W
PLW13 0.08748700 W
F2 - Processing parameters
SI 32768
SF 100.6177975 MHz
WDW EM
SSB 0
LB 2.00 Hz
GB 0
PC 1.40
150 140 130 120 110 100 90 80 70 60 50 40 30 20 10 ppm

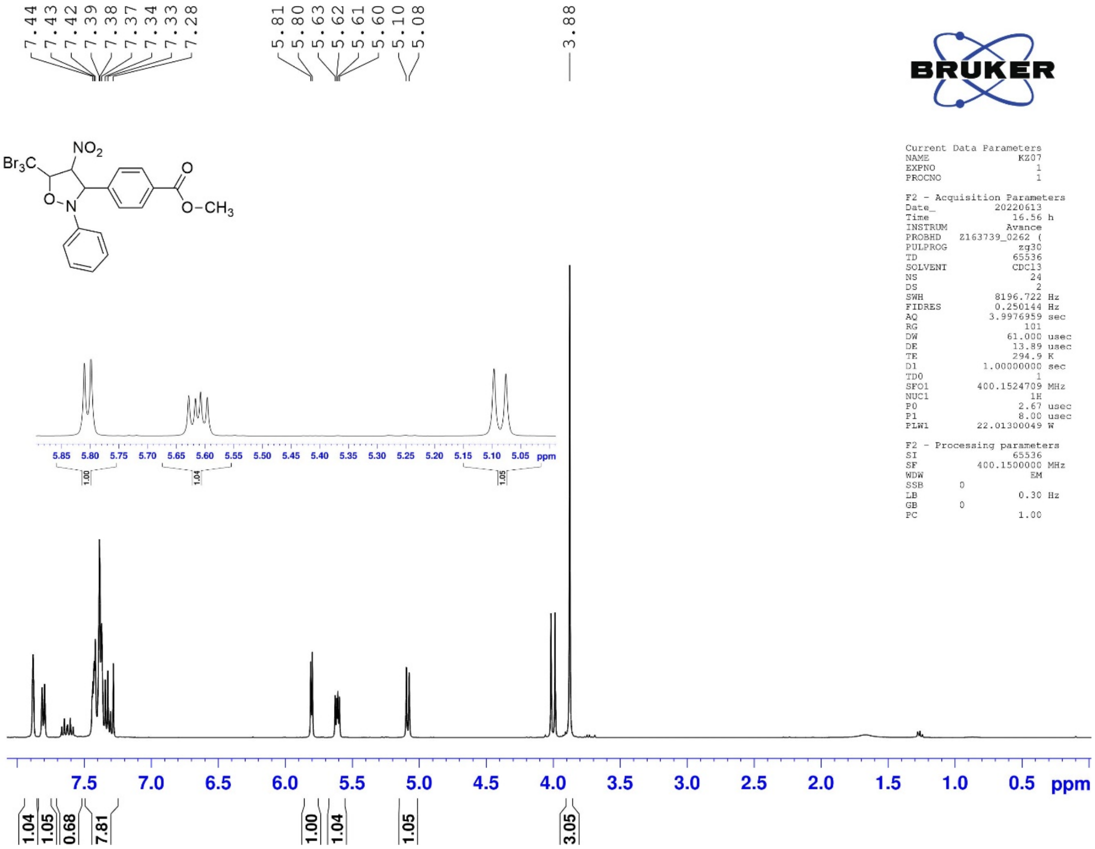
7.44
7.43
7.42
7.39
7.38
7.37
7.34
7.33
7.28
5.81
5.80
5.63
5.62
5.61
5.60
5.10
5.08
3.88
BRUKER
Current Data Parameters
NAME KZ07
EXPNO 1
PROCNO 1
F2 - Acquisition Parameters
Date_ 20220613
Time 16.56 h
INSTRUM Avance
PROBHD Z163739_0262 (
PULPROG zg30
TD 65536
SOLVENT CDCl3
NS 24
DS 2
SWH 8196.722 Hz
FIDRES 0.250144 Hz
AQ 3.9976959 sec
RG 101
DW 61.000 usec
DE 13.89 usec
TE 294.9 K
D1 1.00000000 sec
TD0 1
SFO1 400.1524709 MHz
NUC1 1H
P0 2.67 usec
P1 8.00 usec
PLW1 22.01300049 W
F2 - Processing parameters
SI 65536
SF 400.1500000 MHz
WDW EM
SSB 0
LB 0.30 Hz
GB 0
PC 1.00
5.85 5.80 5.75 5.70 5.65 5.60 5.55 5.50 5.45 5.40 5.35 5.30 5.25 5.20 5.15 5.10 5.05 ppm
1.00 1.04 1.05
7.5 7.0 6.5 6.0 5.5 5.0 4.5 4.0 3.5 3.0 2.5 2.0 1.5 1.0 0.5 ppm
1.04 1.05 0.68 7.81 1.00 1.04 1.05 3.05

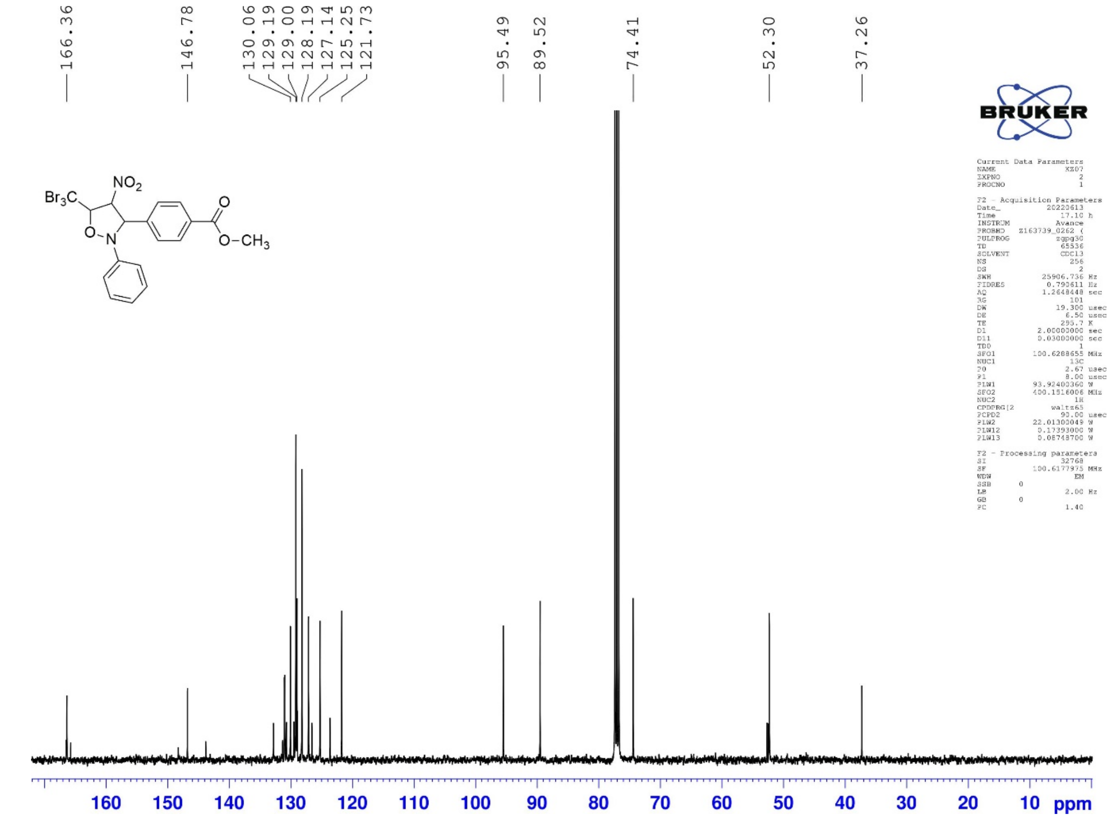

Table S1. The *m/z* values and a molecular formula of the major fragment ions in APCI mass spectra of **4a**-**4g**.

	**[M-NO$_2$+H$^+$]$^+$**	**[M-CBr$_3$+H$^+$]$^+$**	**[M-C$_2$HOBr$_3$+H$^+$]$^+$**	**[M-CNO$_2$Br$_3$+H$^+$]$^+$**	**[M-C$_3$H$_2$NO$_2$Br$_3$+H$^+$]$^+$**	**[M-C$_3$H$_2$NO$_2$Br$_3$+H$^+$]$^+$**
**4a**	502.8729 ($C_{17}H_{16}NO_2Br_3$)	300.1111 ($C_{16}H_{16}N_2O_4$)	271.1083 ($C_{15}H_{15}N_2O_3$)	254.1179 ($C_{16}H_{16}NO_2$)	228.1024 ($C_{14}H_{14}NO_2$)	212.1076 ($C_{14}H_{14}NO$)
**4b**	486.8782 ($C_{17}H_{16}NOBr_3$)	284.1163 ($C_{16}H_{16}N_2O_3$)	255.1129 ($C_{15}H_{15}N_2O_2$)	238.1226 ($C_{16}H_{16}NO$)	212.1069 ($C_{14}H_{14}NO$)	196.1126 ($C_{14}H_{14}N$)
**4c**	472.8630 ($C_{16}H_{14}NOBr_3$)	270.1006 ($C_{15}H_{14}N_2O_3$)	241.0980 ($C_{14}H_{13}N_2O_2$)	224.1075 ($C_{15}H_{14}NO$)	198.0920 ($C_{13}H_{12}NO$)	182.0970 ($C_{13}H_{12}N$)
**4d**	490.8524 ($C_{16}H_{13}NOBr_3F$)	288.0909 ($C_{15}H_{13}N_2O_3F$)	259.0872 ($C_{14}H_{12}N_2O_2F$)	242.0978 ($C_{15}H_{13}NOF$)	216.0823 ($C_{13}H_{11}NOF$)	200.0871 ($C_{13}H_{11}NF$)
**4e**	506.8232 ($C_{16}H_{13}NOBr_3Cl$)	304.0616 ($C_{15}H_{13}N_2O_3Cl$)	275.0590 ($C_{14}H_{12}N_2O_2Cl$)	258.0682 ($C_{15}H_{13}NOCl$)	232.0526 ($C_{13}H_{11}NOCl$)	216.0578 ($C_{13}H_{11}NCl$)
**4f**	550.7731 ($C_{16}H_{13}NOBr_4$)	348.0114 ($C_{15}H_{13}N_2O_3Br$)	319.0083 ($C_{14}H_{12}N_2O_2Br$)	302.0183 ($C_{15}H_{13}NOBr$)	276.0023 ($C_{13}H_{11}NOBr$)	260.00074 ($C_{13}H_{11}NBr$)
**4g**	532.88674 ($C_{18}H_{16}NO_3Br_3$)	328.1048 ($C_{17}H_{16}N_2O_5$)	299.1032 ($C_{16}H_{15}N_2O_4$)	282.1117 ($C_{17}H_{16}NO_3$)	256.0974 ($C_{15}H_{14}NO_3$)	240.1025 ($C_{15}H_{14}NO_2$)
